# Asymmetry of Height and Width of Ethmoid Sinus and Its Association with Chronic Sinusitis: A Cross Sectional Study

**DOI:** 10.31661/gmj.v9i0.1748

**Published:** 2020-07-07

**Authors:** Mehrnoosh Mousaviagdas, Nikzad Shahidi, Shima Majidi, Zhila Khamnian

**Affiliations:** ^1^ENT Department, Faculty of Medicine, Tabriz University of Medical Sciences, Tabriz, Iran; ^2^Social Medicine Department, Faculty of Medicine, Tabriz University of Medical Sciences, Tabriz, Iran

**Keywords:** Ethmoid Sinus, Sinusitis, Ethmoid Sinusitis

## Abstract

**Background:**

In this study, we aimed to investigate the association between chronic anterior sinusitis with the width and height of ethmoid sinus and also assess the ethmoid length and roof asymmetry in the Iranian adult population. **Material and Methods:** This cross-sectional study was carried out on 422 patients who were referred with clinical signs of rhinosinusitis to the ENT Center of Tabriz University of Medical Sciences. Study participants were divided into healthy and sinusitis groups based on the level of sinus involvement. Computed tomography images were applied to calculate ethmoid height and width. A paired t-test was used to assess the roof and width asymmetry and an independent t-test was used to investigate the association between ethmoid height and width with the incidence of rhinosinusitis.

**Results:**

The mean age of sinusitis and healthy groups was 42.5±18.9 and 38.4±17.1 years, respectively. Of a total 422 subjects, 63.4% of whom were men. The overall prevalence of rhinosinusitis was 28.0%. We observed a statistically significant difference in terms of ethmoid height, and in both healthy and sinusitis group right ethmoid roof was statistically lower (P<0.05). However, no statistical difference was observed between the left and right side regarding the ethmoid sinus width (P>0.05). We also estimated correlation coefficients for rhinosinusitis score and ethmoid sinus height and width, which were not statistically significant (P>0.05).

**Conclusion:**

Our study shows that the right side of the ethmoid roof was lower in comparison to the left side, and it should be fully understood and regarded in rhino sinus surgery. We also observed no association between sinusitis score and height and width of the ethmoid sinus.

## Introduction

Rhinosinusitis is one of the most prevalent chronic disease, which is known with more than 3-month mucosal inflammation in nose and sinuses. The prevalence of rhinosinusitis has reported 10-15% in USA adults, and the highest prevalence was observed in 30-60 years [1,2]. Therapeutic options for rhinosinusitis vary from drug-based treatment to rhino surgery in patients who were not treated despite receiving appropriate medicine. Developing countries such as Iran are now facing with rhinosinusitis as a health challenge. Amiri *et al*. reported a pretty high prevalence of rhinosinusitis in Iran [3]. According to their study, more than 50% of Iranian adults suffer from rhinosinusitis that is considerably higher than in developed countries. Treatment of rhinosinusitis imposes a considerable cost on both patients and the health system. Therefore, it is essential to fully understand its etiology to find the best approach for the treatment of rhinosinusitis [4]. In previous studies depicted that size and airing of sinuses are the main factors in the etiology of sinusitis [5,6]. Ethmoid sinus has the most complicated structure, and there are numerous normal variations of ethmoid sinus [7]. Ethmoid sinus mostly involves in inflammatory processes, and most re-operations are attributed to this sinus [7,8]. The associations between ethmoid sinus variation such as Haller cells, deviation of the septum, and very large ethmoid bulla, have been already investigated [9]. However, there is a lack of evidence in terms of the association between height and width of ethmoid sinus and incidence and rhinosinusitis. Considering the high prevalence of rhinosinusitis and the high cost of rhinosinusitis treatment, knowing ethmoid sinus pathology and its alteration may lead to an increase in the efficacy of rhinosinusitis treatments and reduced the surgery-associated complications. In the current study, we aimed to investigate the association between ethmoid sinus volumetric index and incidence of chronic anterior rhinosinusitis, and ethmoid sinus asymmetry in the Iranian population.

## Materials and Methods

**Figure 1:**
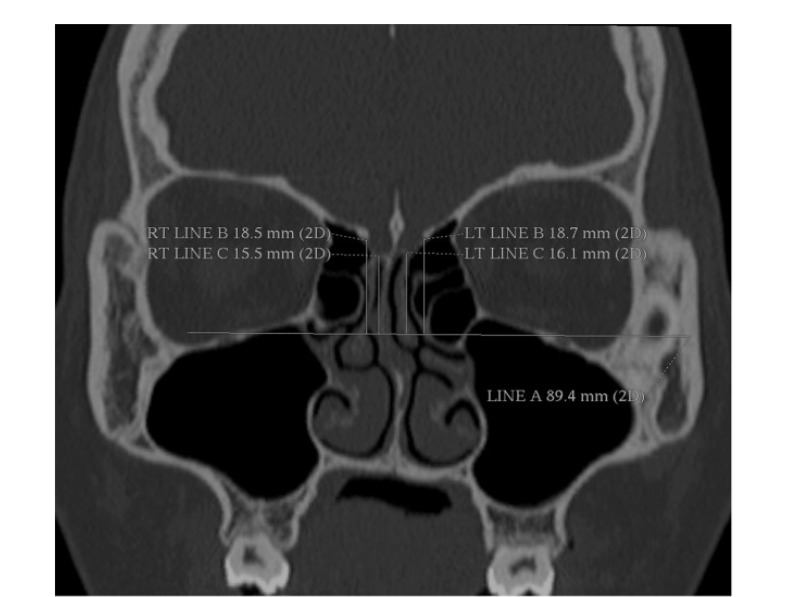
Coronary CT scan of a patient with chronic sinusitis and applied line A, B and C to calculate left and right ethmoid roof’s height and width

 This cross-sectional study was performed on patients who were admitted at the ENT clinic of Tabriz University of Medical Sciences with clinical signs of chronic anterior sinusitis. Study participants had symptoms of sinusitis at least for three months, and computed tomography (CT) images at Coronal, Axial, and Sagittal dimensions were available for them, which were captured in one of the radiography centers affiliated to Tabriz University Medical of Sciences. The exclusion criteria were age under 18 years, having other rhinosinusitis (such as allergic or fungal rhinosinusitis), previous surgery of sinus, history of major head trauma, and CT scans with anatomical variations (such as septum deviation, very big ethmoid, Haller cell, and paradoxical turbine). A high-resolution CT scan was used to assess CT images. As it is shown in Figure-1, we draw three lines to calculated ethmoid height. Line A was a horizontal line between two lower orbital holes. Line B was a direct vertical line connecting to line A at the junction of the lateral lamella of the cribriform plate (LLCP) and fovea ethmoidal. Line C was another vertical line perpendicular to line A at the connection place of the cribriform plate to LLCP. Ethmoid height was a subtraction of length of line C and line B (Figure-1). We used the Lund Mackay scoring system to score sinusitis involvement. In this scale, we used the following instruction: no involvement=0, partial involvement=1, and total involvement=2. Overall sinusitis score was the sum of maxillary, frontal, anterior ethmoid, and osteomeatal complex sinuses scores that ranged from 0 to 8. Sinusitis patients were divided into three groups according to their sinusitis score. Scores 1 and 2 were minor sinusitis, scores 3, 4, and 5 were grouped as moderate sinusitis, and the major sinusitis group was patients with scores 6-8. We also considered the space between orbit and junction of LLCP and fovea ethmoidalis as the width of the ethmoid. Two ENT specialists have done all measurements

 Descriptive analysis was applied to show the distribution of both continuous and dichotomous variables. We also used a paired t-test to assess the difference between right and left ethmoid width and height. Also, Chi-square and independent t-test were used to compare the investigated variables between healthy and sinusitis groups. Besides, we used Pearson correlation coefficients to assess the correlation between ethmoid height and width with sinusitis involvement score. All statistical analysis was performed using Stata software (Ver 14.1, Collage Station, Texas, USA).

## Results

 This study was carried out on 424 patients who were referred to the ENT clinic of Tabriz University of Medical Sciences with sinusitis symptoms. Study participants were divided into sinusitis and healthy control groups according to the sinusitis score. The mean age of the sinusitis group was 42.5±18.9 years, while healthy individuals were statistically younger (38.4±17.1years). Overall, 64.3% of study participants were men. The prevalence of anterior sinusitis in the study participants was 28.0%. In the right sinus, 341 of study participants were healthy (80.4%), 4.2% had minor sinusitis, and the percentage of moderate and major sinusitis was 9.2% and 6,1%, respectively. Accordingly, for left sinus minor sinusitis was observed in 5,1% and 9.9% of study participants had moderate sinusitis. Also, major sinusitis noted in 7.0 of participants (Table-1). Mean of ethmoid sinus height and width for healthy group, minor, moderate and major sinusitis groups was reported in Table-2, and no statistical difference was found in both left and the right side between compared groups. Mean of right and left ethmoid sinus height in healthy group was 4.5±1.8 and 4.7±1.8, respectively, and the observed difference was statistically significant (P=0.037, Table-3). Also, a statistical difference was observed between left and right ethmoid sinus height in sinusitis patients where the mean of left ethmoid sinus height was 4.8±2.1, and it was 4.4±1.8 for right ethmoid sinus (P=0.006, Table-2). In 30.1% of study participants, the left ethmoid roof was higher, 20.5% had a higher roof on the right side, and the symmetric ethmoid roof was observed in 49.2% (Table-4). Comparison of left and right ethmoid sinus width in both healthy and sinusitis groups depicted no statistical difference (P-value>0.05, Table-3). We also observed left width asymmetry in 29.7%, 25.2% right asymmetry, and 45.0% of study participants right and left ethmoid width were equal; however, there was no statistical difference between healthy and sinusitis groups (P=0.877, Table-4).

## Discussion

The current study assessed the roof and width asymmetry and correlation with chronic anterior rhinosinusitis among patients who were referred to the ENT clinic of Tabriz University of Medical Sciences. We used CT images as an essential tool to investigate pathological changes and anatomic abnormalities of the nose and paranasal sinus. It is depicted in previous studies CT images could be used either as a prognostic tool or a simple method to assess ethmoid roof asymmetry and sinus involvement. In the current study, we compared left and right ethmoid roof height and observed that in both healthy and sinusitis group right ethmoid had a lower roof. We found that 224 cases (52.8%) had a lower ethmoid roof on the right side, and it was 40.5% on the left. Previous studies support our findings, and it is depicted that the right ethmoid has a lower roof. Reib *et al*. reported higher asymmetry in the right ethmoid than left, which is in line with our findings [10]. The same results have been reported by Lebowitz *et al*. [11]. Despite previous findings that reported higher asymmetry among women than men, we observed no statistical difference in this regard [10]. However, there are some other studies in which no association was observed between ethmoid height and sex [12]. In our study, the prevalence of ethmoid roof symmetry was estimated at 49.2%. While, in previous studies, it is reported between 40-70%. Racial differences are the main variable that could justify the different prevalence of ethmoid roof asymmetry in previous researches [13]. We also determined asymmetry in the lateral lamina. Overall, 233 study participants had lateral lamina asymmetry. Less than 50.0% lateral lamella asymmetry has been reported in several studies, and it seems this asymmetry may lead to surgical difficulties, as it is related to flattening of the fovea ethmoidalis and angulation of the LLCP [14,15]. In 29.7% of our participants, the left side was longer, while in 25.2%, it was vice versa. However, no statistical difference was observed between sinusitis and healthy groups. Few studies compared left and right lateral lamella asymmetry. Bollain *et al*. reported a higher percentage of asymmetry on the right side than the left one, which is in contrast to our findings. Bollain *et al*. reported a longer left side of 39 patients, whereas 61 patients had the longer right side. We also observed no statistical difference between men and women in terms of lateral lamella width, although it was partially higher among women (59.0%) than men (52.8%). Our result was confirmed by Bollain *et al*. as they did not report any association between lateral lamella asymmetry and sex [16]. We also assessed the relationship between sinusitis score involvement and ethmoid sinus height and width. There was no association between the width and height of ethmoid sinus and the incidence of chronic anterior rhinosinusitis. In the literature review, there were few studies in which the effect of ethmoid height and width was assessed regarding the incidence of rhinosinusitis. However, Pashaoglu *et al*. reported no association between risk of ethmoid sinusitis and the height and width of ethmoid sinus [17]. The retrospective approach was the main limitation of our study, which can affect our findings. Also, patients under 18-year old were excluded from the study. However, the current study is one of the few studies in which the association between height and width of ethmoid sinus and incidence of rhinosinusitis.

## Conclusion

 The present study highlighted the anatomic variation of ethmoid sinus, particularly variation in the ethmoid roof, which is related to a major complication of endoscopic sinus surgery. It could be concluded that the right side of the ethmoid roof was lower in comparison to the left side, and it should be fully understood and regarded in rhino sinus surgery. Moreover, there was no association between height and width of ethmoid sinus and the risk of rhinosinusitis. However, more researches are required.

## Acknowledgment

 The Tabriz University of Medical Sciences supported this research. We sincerely thank all our colleagues from the ENT clinic of Tabriz University Medical of Sciences who helped us in data gathering.

## Conflict of Interest

 All authors declare that they have no conflict of interests.

**Table 1:** Number of Healthy, Minor, Moderate, and Major Sinusitis Patients for Right and Left Sinuses

**Variable**	**Healthy**	**Minor Sinusitis**	**Moderate Sinusitis**	**Major Sinusitis**
**Right sinus**	341 (80.4%)	18 (4.2%)	39 (9.2%)	26 (6.1%)
**Left sinus**	330 (77.8%)	22 (5.1%)	42 (9.9%)	30 (7.0%)

**Table 2:** Mean (±SD) of Sinus Height and Width in Healthy, Minor, Moderate, and Major Sinusitis Groups

**Variables**	**Healthy**	**Minor Sinusitis**	**Moderate Sinusitis**	**Major Sinusitis**	**P-value**
**Right sinus height**	4.6 (±1.8)	5.2 (±2.5)	4.6 (±1.4)	4.4 (±1.6)	0.095
**Right sinus width**	7.6 (±1.6)	8.0 (±1.6)	7.4 (±1.6)	7.5 (±1.2)	0.598
**Left sinus height**	4.7 (±1.8)	5.3 (±2.9)	4.7 (±2.0)	4.8 (±1.8)	0.087
**Left sinus width**	7.6 (±1.7)	8.1 (±2.1)	7.7 (±1.5)	7.9 (±1.3)	0.588

**Table 3:** Ethmoid Sinus Asymmetry Assessment in Rhinosinusitis and Healthy Groups

**Variable **	**Sinusitis Group**			**Healthy Group**		
	Left side	Right side	P-value	Left side	Right side	P-value
Ethmoid height	4.8 (2.1)	4.4 (1.8)	0.006	4.7 (1.8)	4.5 (1.8)	0.037
Ethmoid width	7.7 (1.6)	7.6 (1.5)	0.342	7.7 (1.7)	7.6 (1.7)	0.423

**Table 4:** Distribution of Height and Width of the Ethmoid Rregarding Asymmetry and Symmetry Status in The Study Participants by Sinusitis Status

**Variables**	**Sinusitis Group **n (%)	**Healthy Group **n (%)
Lower left ethmoid roof	18 (15.1%)	69 (22.6%)
Lower right ethmoid roof	42 (35.2%)	86 (28.2%)
Symmetric roof	59 (49.5%)	150 (49.1%)
Wider right lateral lamella	32 (26.8%)	75 (24.5%)
Wider left lateral lamella	34 (28.5%)	92 (30.1%)
